# Supplementary material for: Application of a Biphasic Mathematical Model of Cancer Cell Drug Response for Formulating Potent and Synergistic Targeted Drug Combinations to Triple Negative Breast Cancer Cells
Source: Cancers (Basel). 2020 Apr 27;12(5):1087. doi: 10.3390/cancers12051087 (PMC7281712; doi:10.3390/cancers12051087)
Supplement: Supplementary file 1 [file cancers-12-01087-s001.zip › Revised Figure S1 and Table S1.pdf]

Fig. S1. Full pictures of Western blot analyses of selected kinase inhibitors on MDA-MB-468 and MDA-MB-231 cell line signaling. These are the full pictures of the Western blot results used to generate Fig. 6. The cell line and treatments were shown above the pictures. 1, Control; 2, Lapatinib; 3, GSK690693; 4, Lapatinib+GSK690693; 5, Dasatinib; 6, AZD-6244; 7, Dasatinib+AZD-6244. The concentrations of the drugs and treatment conditions are described in the main paper. The borders for cropping the images to produce the images in Fig. 6 are indicated by the dashed lines.

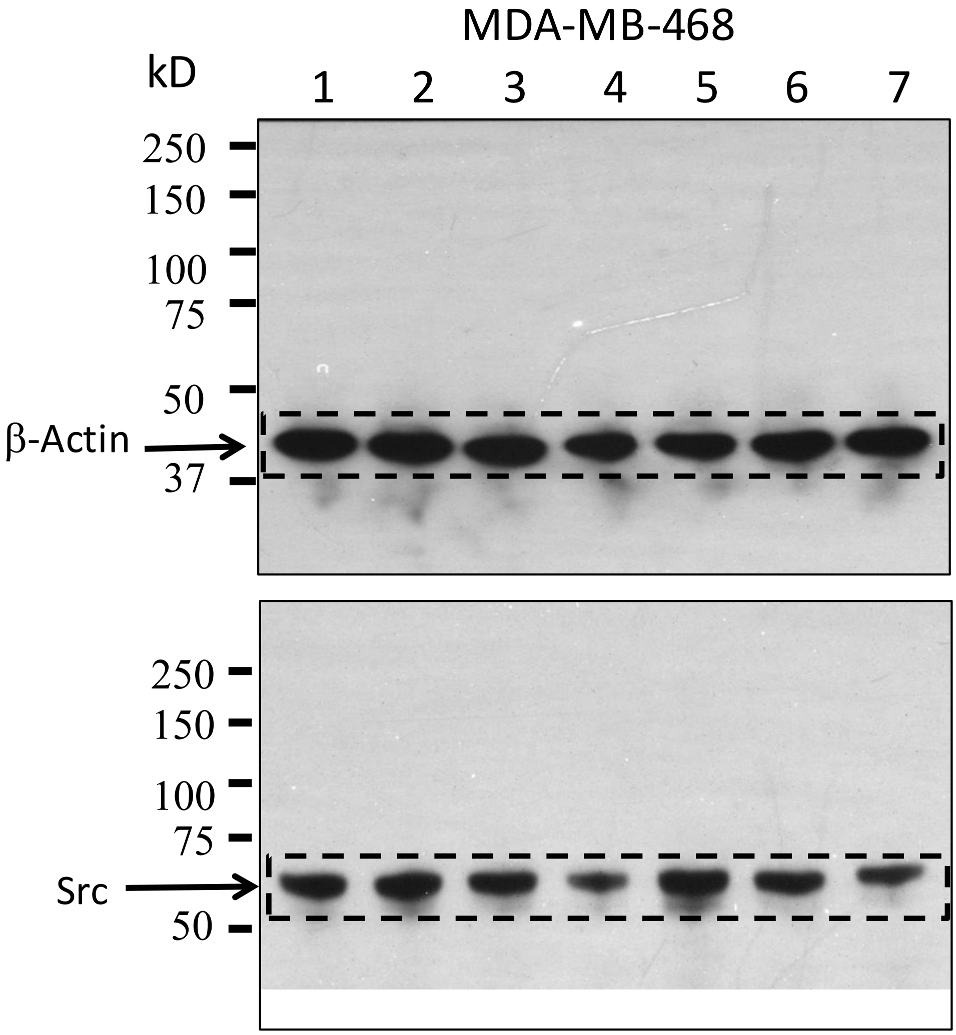

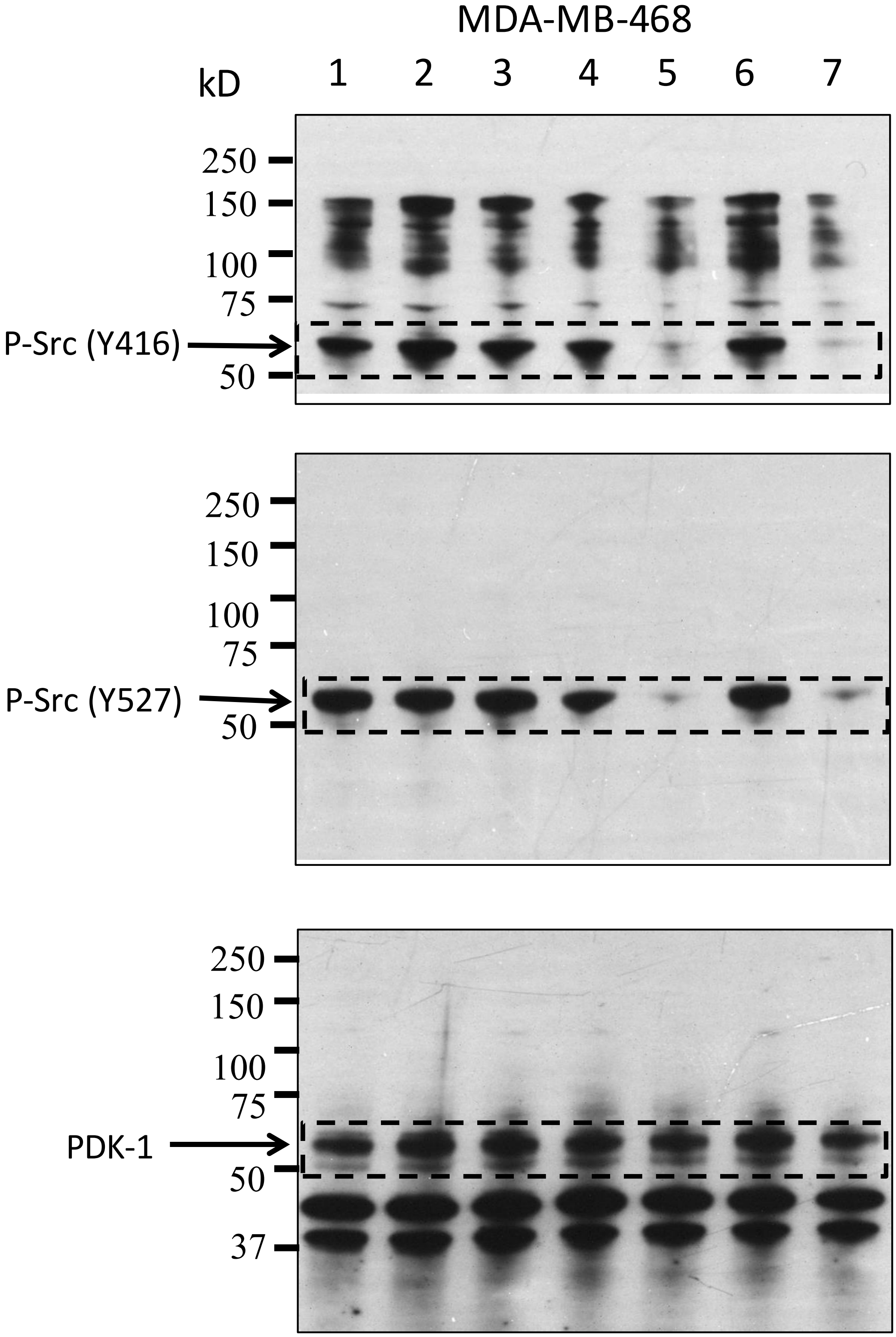
MDA-MB-468
kD
1 2 3 4 5 6 7
250
150
100
75
50
P-Src (Y416)
250
150
100
75
50
P-Src (Y527)
250
150
100
75
50
37
PDK-1

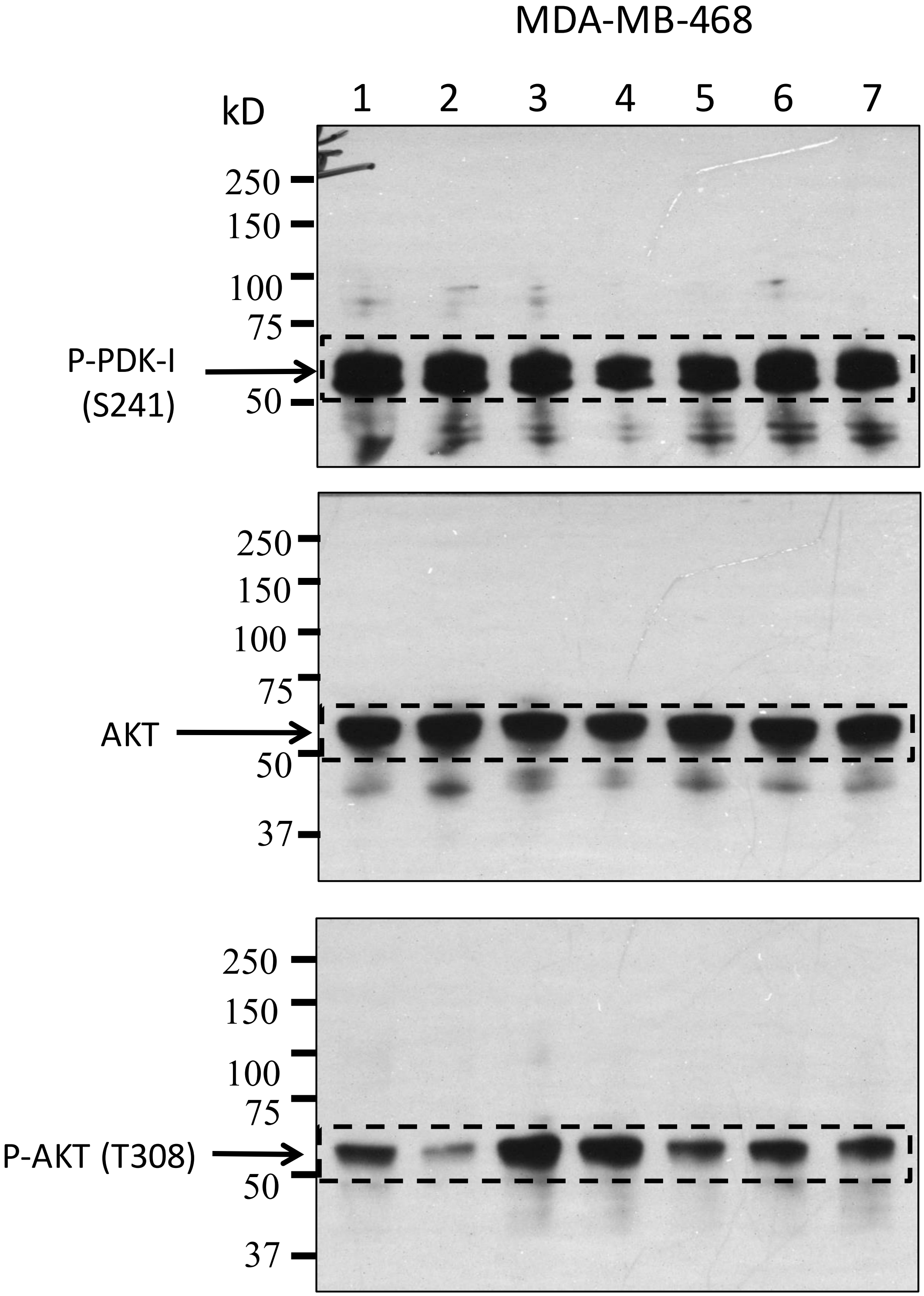
MDA-MB-468
kD
1
2
3
4
5
6
7
250
150
100
75
50
37
P-PDK-I (S241)
AKT
P-AKT (T308)

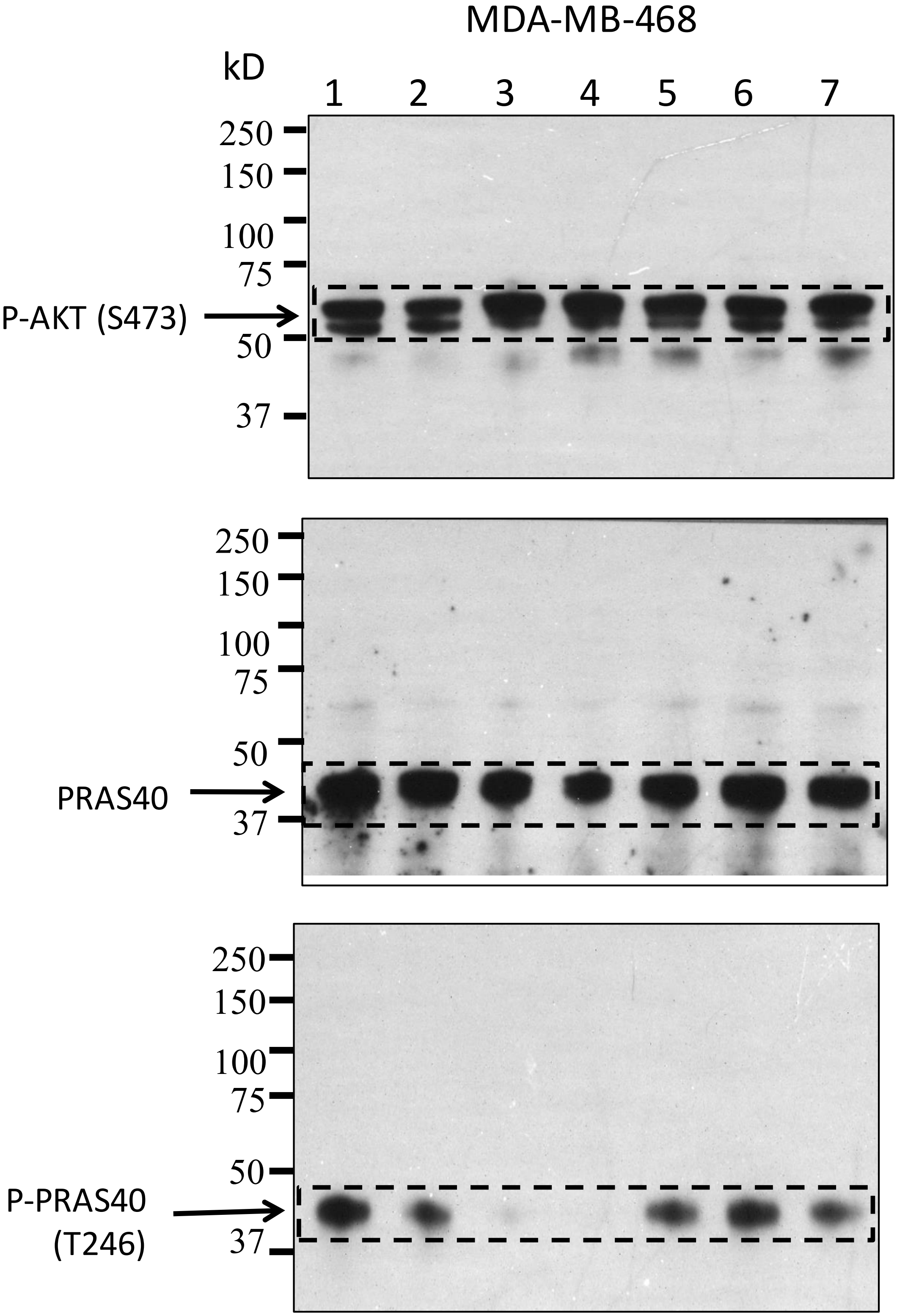
MDA-MB-468
kD
1 2 3 4 5 6 7
250
150
100
75
50
37
P-AKT (S473)
250
150
100
75
50
37
PRAS40
250
150
100
75
50
37
P-PRAS40
(T246)

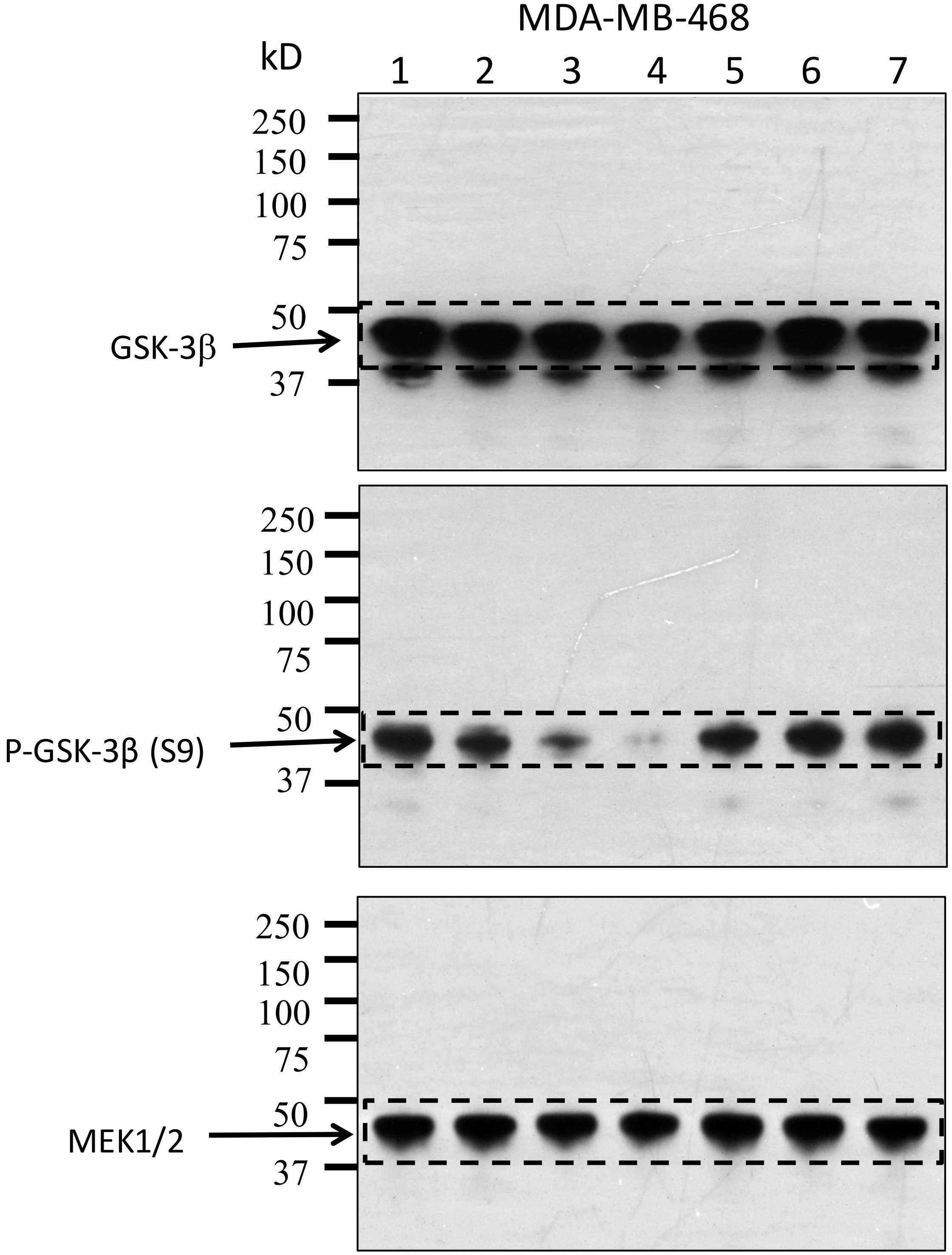
MDA-MB-468
kD
1
2
3
4
5
6
7
250
150
100
75
50
37
GSK-3β
P-GSK-3β (S9)
MEK1/2

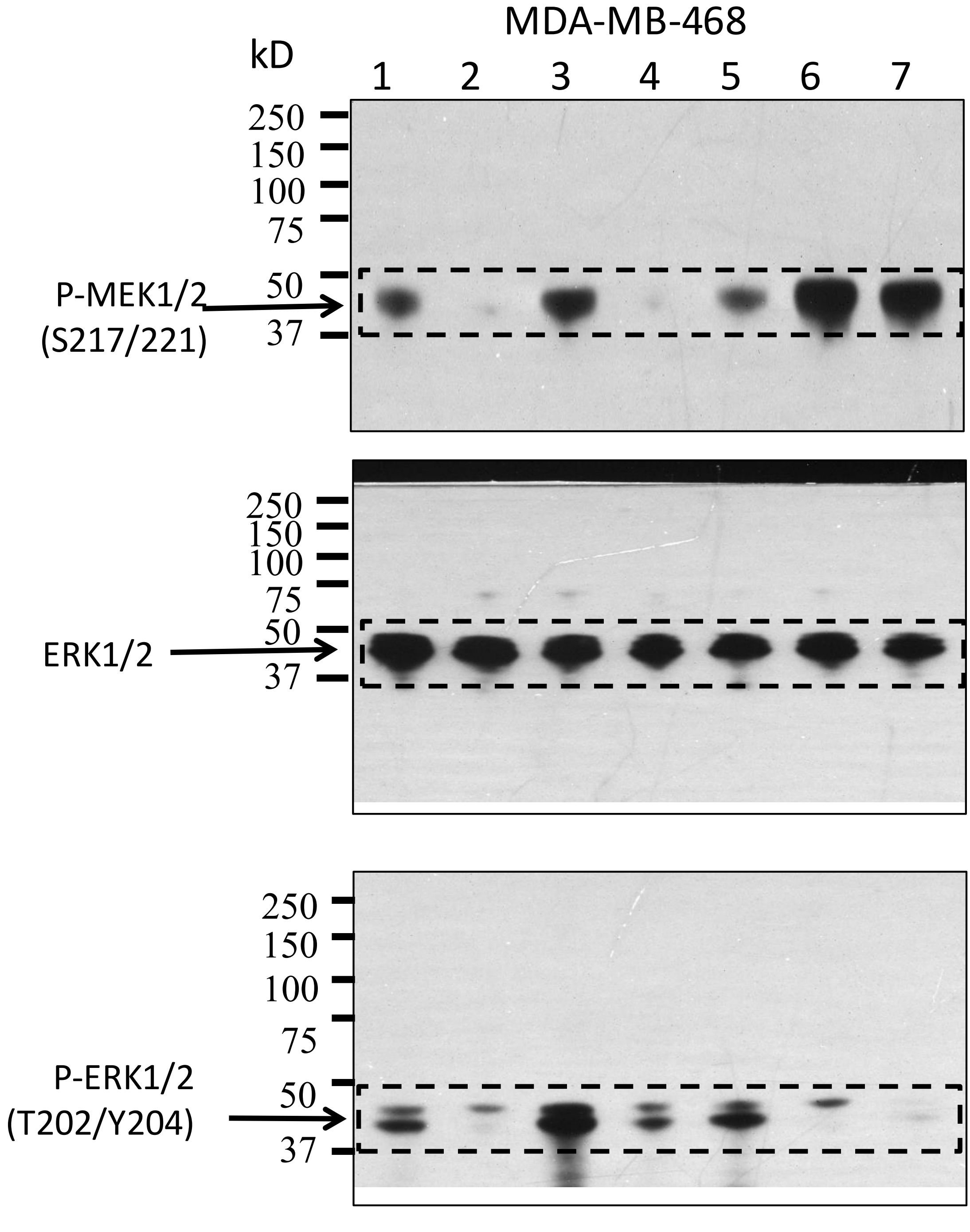

MDA-MB-468
kD
1 2 3 4 5 6 7
250
150
100
75
50
37
P-MEK1/2
(S217/221)
ERK1/2
P-ERK1/2
(T202/Y204)

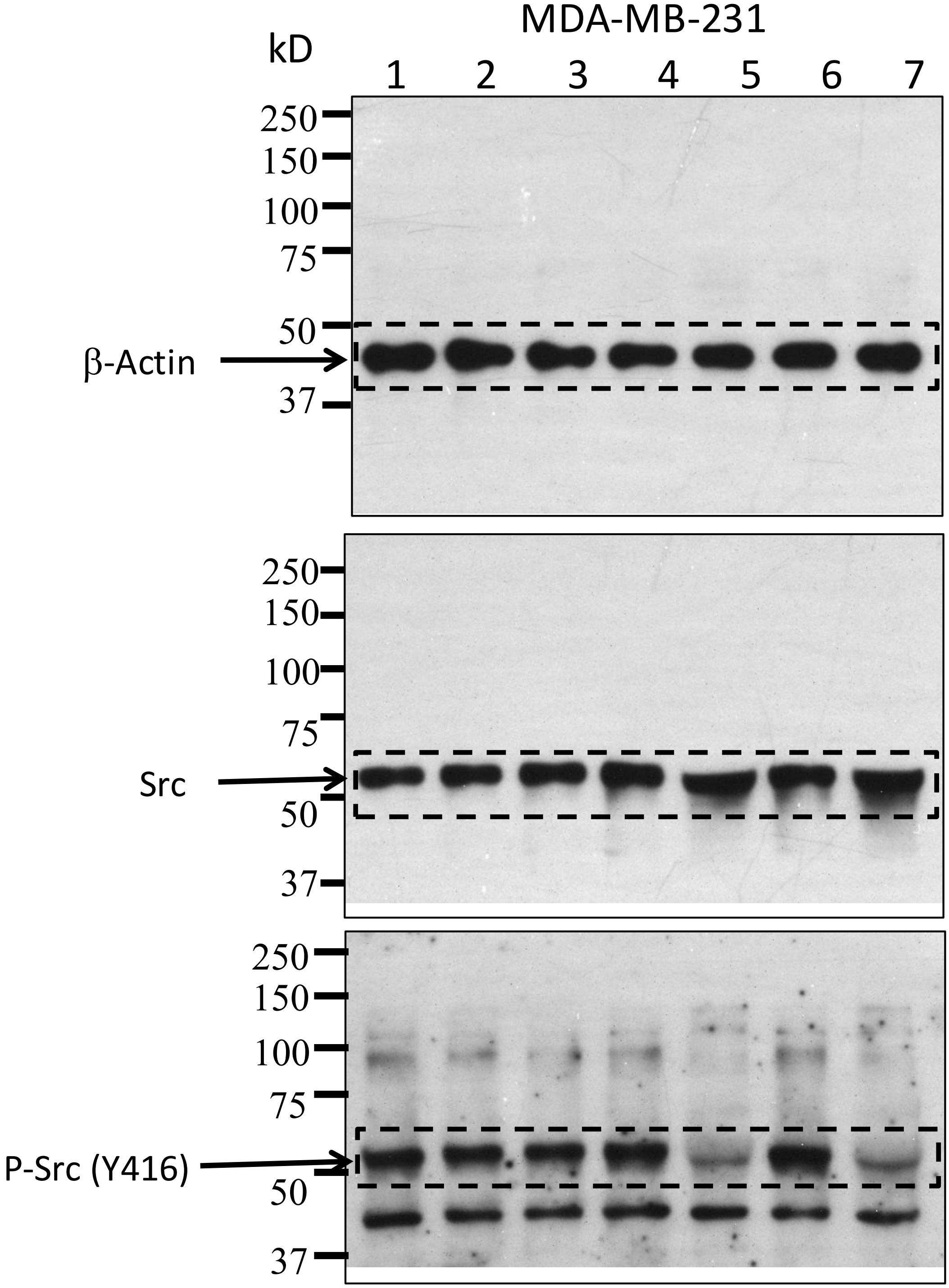
MDA-MB-231
kD
1 2 3 4 5 6 7
250
150
100
75
50
37
β-Actin
250
150
100
75
Src
50
37
250
150
100
75
P-Src (Y416)
50
37

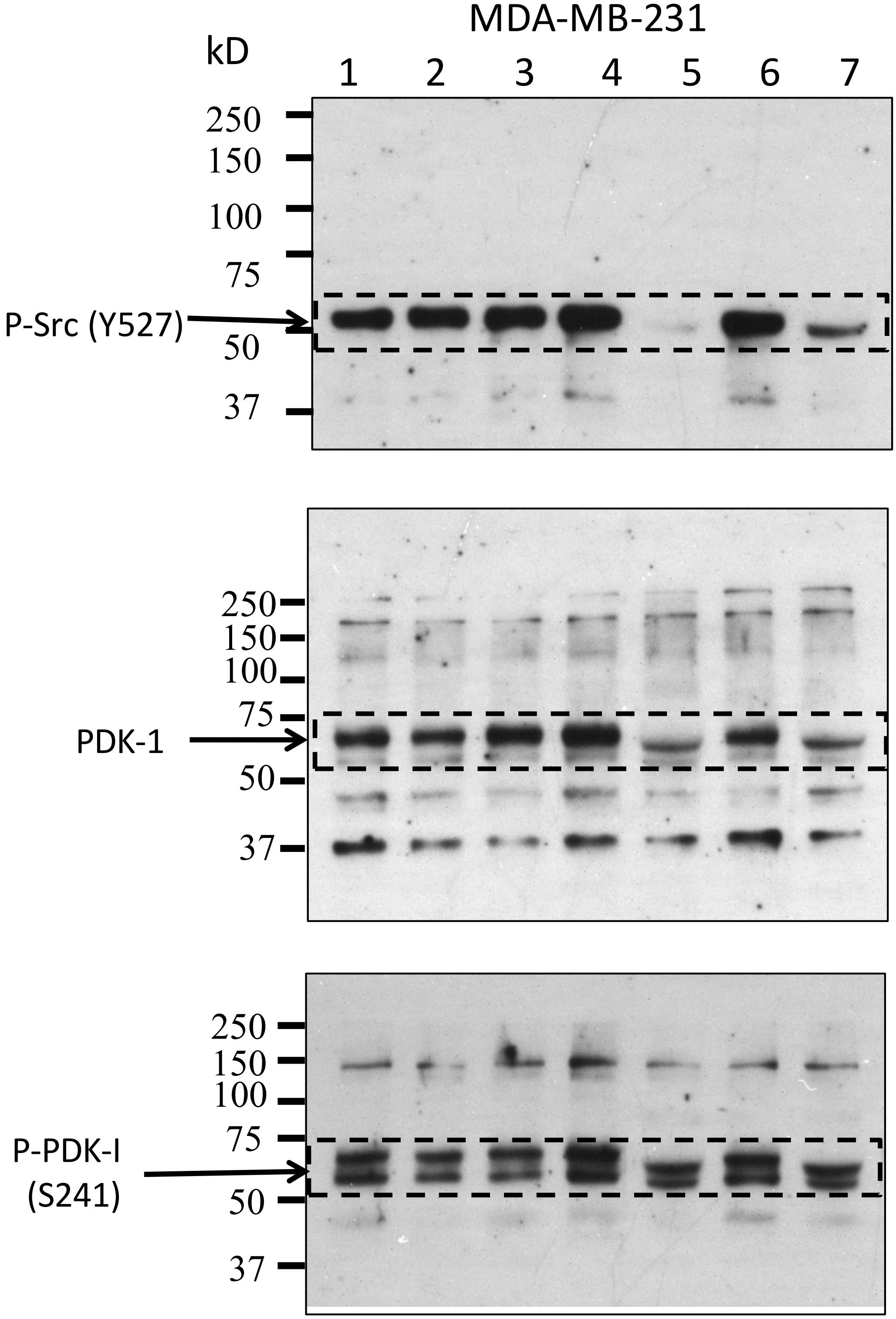
MDA-MB-231
kD
1 2 3 4 5 6 7
250
150
100
75
50
37
P-Src (Y527)
PDK-1
P-PDK-I
(S241)

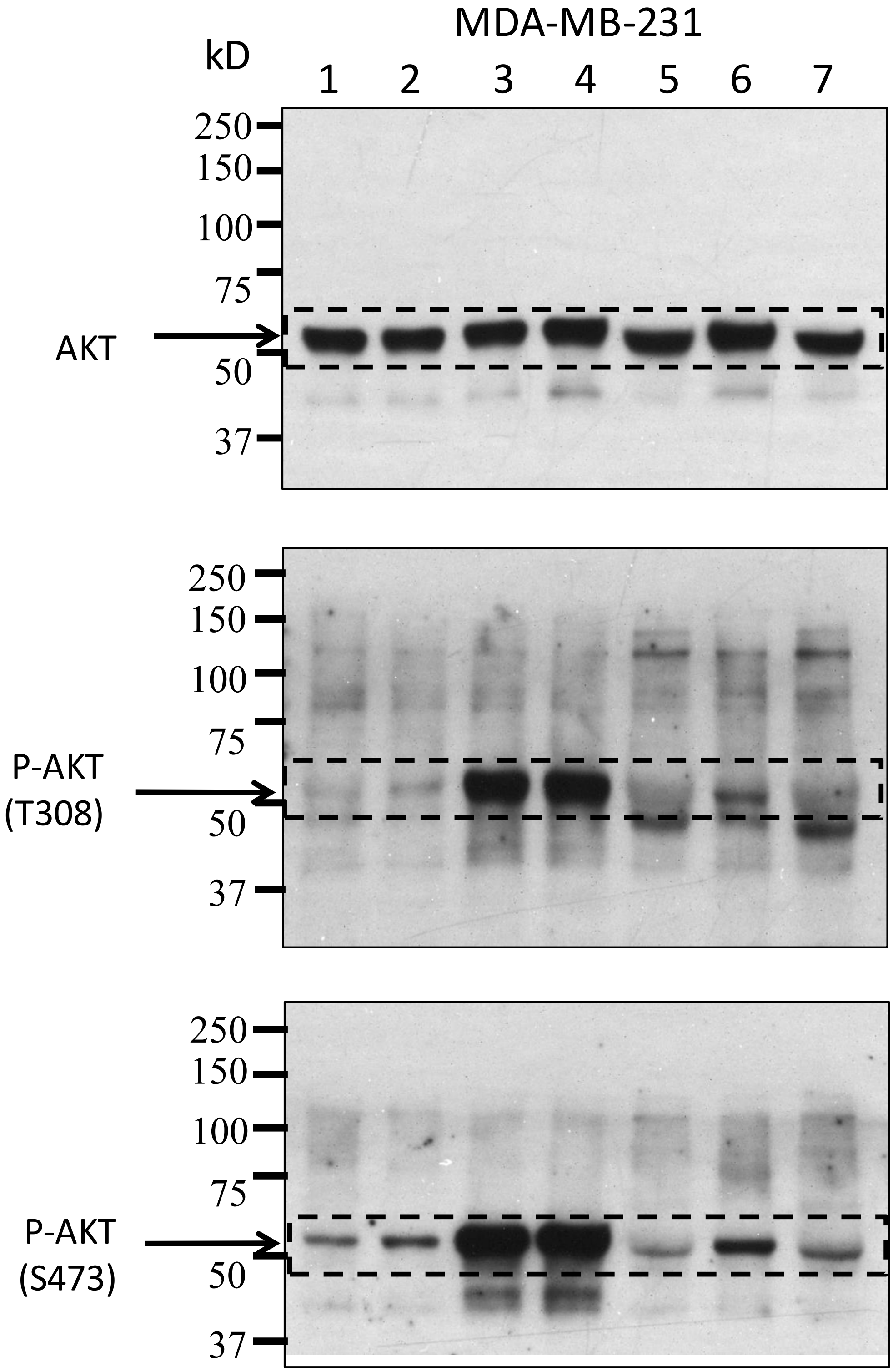
MDA-MB-231
kD
1
2
3
4
5
6
7
250
150
100
75
50
37
AKT
P-AKT
(T308)
P-AKT
(S473)

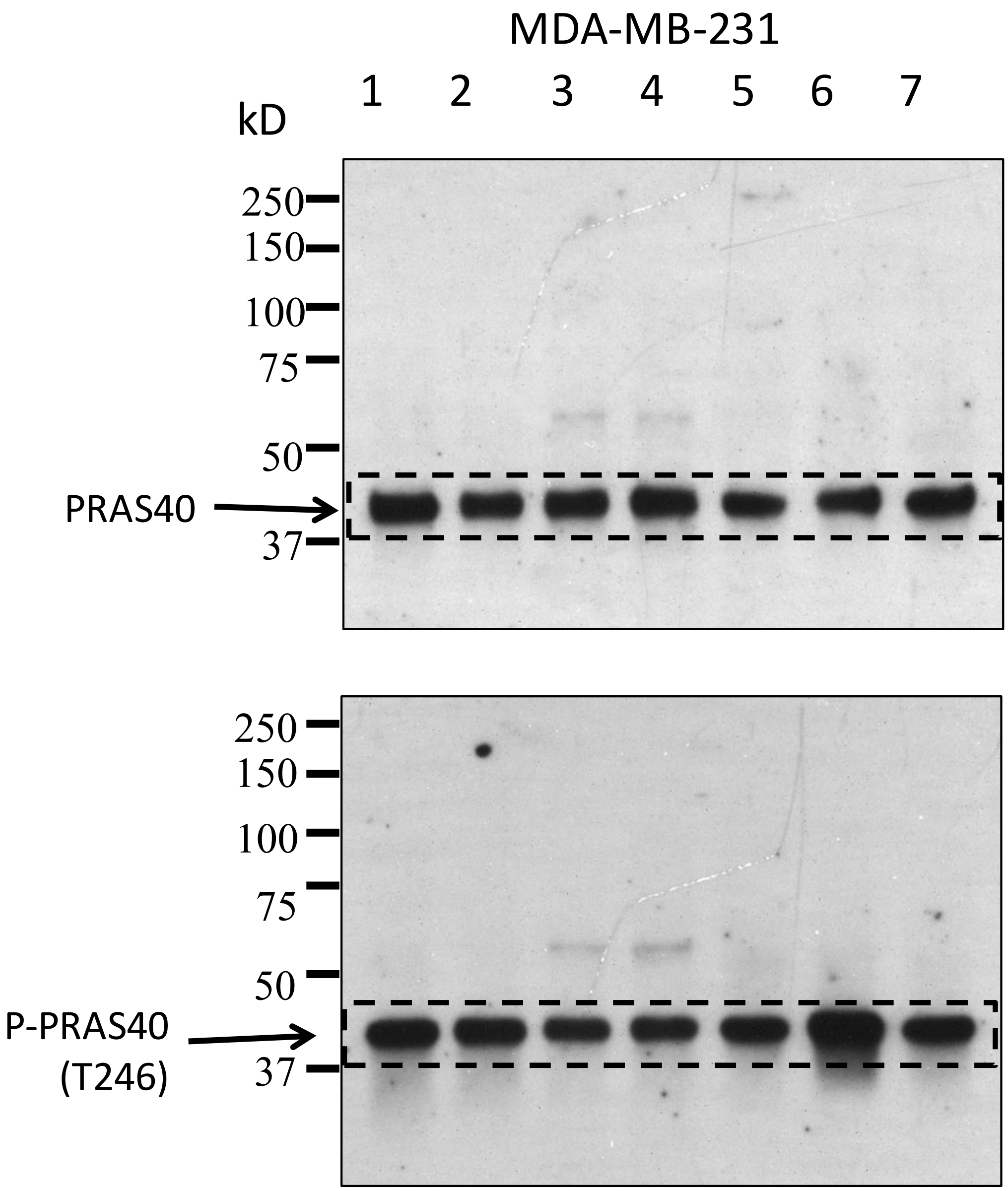
MDA-MB-231
1 2 3 4 5 6 7
kD
250
150
100
75
50
PRAS40
37
250
150
100
75
50
P-PRAS40 (T246)
37

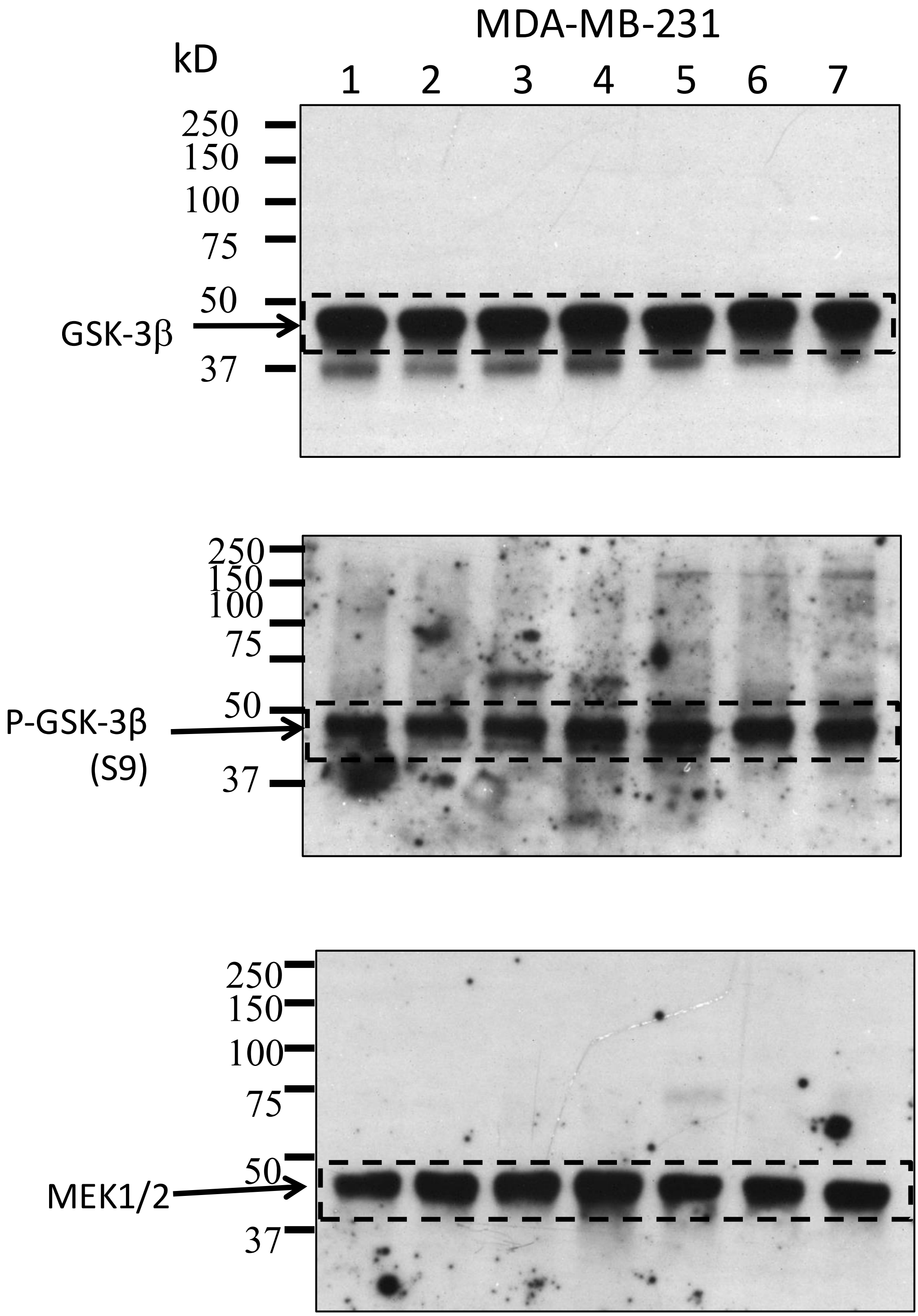
MDA-MB-231
kD
1
2
3
4
5
6
7
250
150
100
75
50
GSK-3β
37
250
150
100
75
50
P-GSK-3β
(S9)
37
250
150
100
75
50
MEK1/2
37

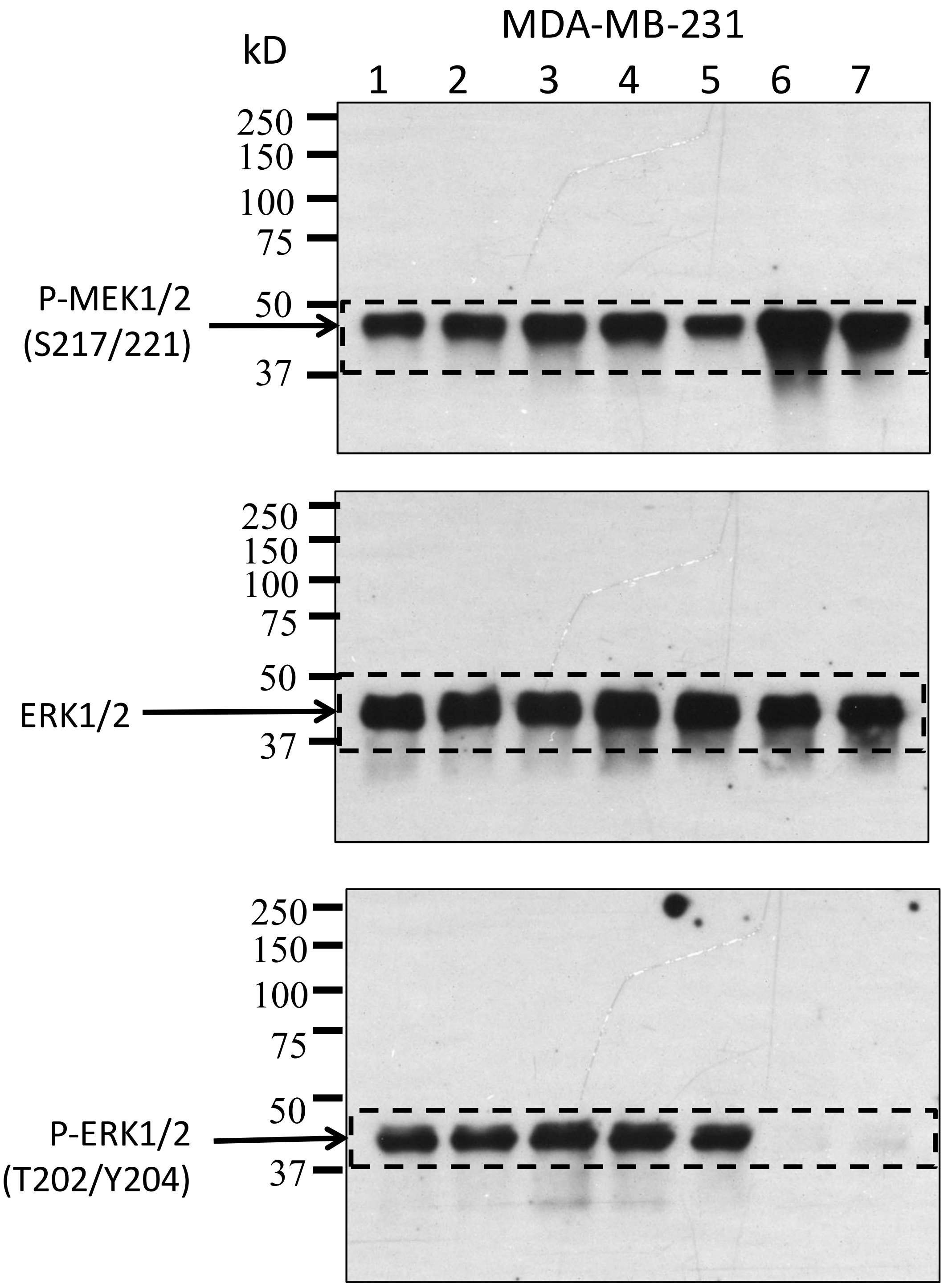
MDA-MB-231
kD
1
2
3
4
5
6
7
250
150
100
75
50
37
P-MEK1/2
(S217/221)
ERK1/2
P-ERK1/2
(T202/Y204)

Table S1. Densitometry analyses of the band density presented in Fig. 6. The densities of appropriate bands in Fig. 6 were analyzed using the ImageJ software. For each protein, the seven bands for all treatments were analyzed using the same analysis box containing the whole band. The densities were compared to the control (no drug treatment) and normalized to the loading control, β-actin. The intensity of the phosphorylated protein bands were also normalized to the level of the same protein. The numbers above 2, suggestive of strong stimulation, are labeled green, and the numbers below 0.5, suggestive of strong inhibition, are labeled red.

MDA-MB-468 (normalized to Control and b-actin)							
	Control	Lapatinib	GSK690693	Lapa+GSK	Dasatinib	AZD-6244	Dasa+AZD
β-Actin	1	1	1	1	1	1	1
Src	1	1.05	1.00	0.81	1.32	0.99	0.82
pSrc-416	1	1.17	0.97	1.00	0.42	1.17	0.28
pSrc-527	1	0.89	1.01	0.91	0.39	1.06	0.39
PDK1	1	1.10	1.20	1.41	1.33	1.23	1.00
pPDK1	1	0.85	0.87	0.79	0.89	0.90	0.84
Akt	1	1.01	1.01	1.03	1.11	1.03	0.99
pAkt-T308	1	0.55	1.45	1.57	1.05	1.09	1.02
pAkt-S473	1	0.85	1.01	1.17	1.07	0.98	1.00
PRAS40	1	0.83	0.70	0.72	0.86	0.90	0.83
pPRAS40	1	0.59	0.21	0.21	0.79	0.88	0.62
GSK-3β	1	0.92	0.95	1.00	1.06	1.00	0.94
pGSK-3β	1	0.80	0.54	0.35	0.99	0.97	1.02
Mek1/2	1	0.96	0.97	1.08	1.21	1.05	1.02
pMek1/2	1	0.44	1.31	0.55	1.00	1.98	1.88
Erk1/2	1	0.86	0.84	0.86	0.86	0.84	0.78
pErk1/2	1	0.42	1.64	0.84	1.28	0.44	0.29

MBA-MB-468 (normalized to protein level)							
	Control	Lapatinib	GSK690693	Lapa+GSK	Dasatinib	AZD-6244	Dasa+AZD
b-Actin							
Src		1	1	1	1	1	1
pSrc-416		1.12	0.97	1.23	0.32	1.18	0.35
pSrc-527		0.85	1.01	1.12	0.29	1.07	0.47
PDK1		1	1	1	1	1	1
pPDK1		0.77	0.73	0.56	0.67	0.73	0.84
Akt		1	1	1	1	1	1
pAkt-T308		0.55	1.44	1.53	0.95	1.06	1.03
pAkt-S473		0.84	1.01	1.14	0.96	0.95	1.02
PRAS40		1	1	1	1	1	1
pPRAS40		0.71	0.30	0.30	0.92	0.99	0.75
GSK-3b		1	1	1	1	1	1
pGSK-3b		0.86	0.57	0.35	0.93	0.97	1.09
Mek1/2		1	1	1	1	1	1
pMek1/2		0.46	1.36	0.51	0.83	1.88	1.84
Erk1/2		1	1	1	1	1	1
pErk1/2		0.49	1.95	0.98	1.49	0.52	0.37

MDA-MB-231 (normalized to Control and b-actin)							
	Control	Lapatinib	GSK690693	Lapa+GSK	Dasatinib	AZD-6244	Dasa+AZD
β-Actin	1	1	1	1	1	1	1
Src	1	1.09	1.35	1.56	1.75	1.59	1.51
pSrc-416	1	1.00	1.12	1.34	0.49	1.31	0.49
pSrc-527	1	1.05	1.31	1.51	0.43	1.37	0.59
PDK1	1	0.98	1.42	1.72	1.00	1.24	0.83
pPDK1	1	0.83	1.06	1.37	1.07	1.19	0.91
Akt	1	1.00	1.20	1.39	1.37	1.44	1.11
pAkt-T308	1	1.08	2.84	3.02	1.83	1.76	1.66
pAkt-S473	1	1.26	3.58	3.84	1.50	1.88	1.56
PRAS40	1	0.86	1.05	1.18	0.99	0.95	1.07
pPRAS40	1	1.03	0.96	1.04	1.21	1.51	1.14
GSK-3β	1	0.98	1.17	1.23	1.31	1.18	1.04
pGSK-3β	1	1.00	1.18	1.30	1.44	1.14	1.14
Mek1/2	1	1.18	1.35	1.59	1.42	1.23	1.21
pMek1/2	1	1.13	1.36	1.50	1.15	1.78	1.55
Erk1/2	1	1.03	1.16	1.35	1.41	1.20	1.20
pErk1/2	1	1.10	1.41	1.44	1.33	0.26	0.25

MDA-MB-231 (normalized to protein level)							
	Control	Lapatinib	GSK690693	Lapa+GSK	Dasatinib	AZD-6244	Dasa+AZD
b-Actin							
Src		1	1	1	1	1	1
pSrc-416		0.92	0.83	0.86	0.28	0.82	0.33
pSrc-527		0.96	0.97	0.97	0.25	0.86	0.39
PDK1		1	1	1	1	1	1
pPDK1		0.85	0.75	0.80	1.07	0.96	1.09
Akt		1	1	1	1	1	1
pAkt-T308		1.08	2.37	2.18	1.34	1.22	1.50
pAkt-S473		1.26	2.99	2.77	1.10	1.30	1.40
PRAS40		1	1	1	1	1	1
pPRAS40		1.19	0.92	0.88	1.22	1.59	1.07
GSK-3b		1	1	1	1	1	1
pGSK-3b		1.02	1.01	1.06	1.10	0.97	1.09
Mek1/2		1	1	1	1	1	1
pMek1/2		0.95	1.00	0.94	0.81	1.44	1.29
Erk1/2		1	1	1	1	1	1
pErk1/2		1.07	1.22	1.07	0.94	0.22	0.21
